# Cultural differences in appraisals of control and posttraumatic stress disorder symptoms

**DOI:** 10.1080/20008066.2024.2358685

**Published:** 2024-06-05

**Authors:** Laura Jobson, Larissa Shiying Qiu, Joshua Wong, Haoxiang Li, July Lies, Winnie Lau, Richard A. Bryant, Belinda J. Liddell

**Affiliations:** a Turner Institute for Brain and Mental Health and School of Psychological Sciences, Monash University, Clayton, Australia; b School of Psychology, University of New South Wales, Sydney, Australia; c Phoenix Australia-Centre for Posttraumatic Mental Health and Department of Psychiatry, University of Melbourne, Carlton, Australia; d School of Psychological Sciences, University of Newcastle, University Drive, Callaghan, Australia

**Keywords:** PTSD, appraisals, Australian, Chinese, self-construal, TEPT, evaluaciones, australianos, chinos, autoconcepto

## Abstract

**Background:** Appraisals are central to posttraumatic stress disorder (PTSD). Yet, few studies have examined how culture influences the associations between different types of trauma-related appraisals and PTSD symptoms.

**Objective:** This study investigated cultural influences on appraisals of control and their associations with PTSD symptoms.

**Method:** European Australian (*n *= 140, *M*age = 35.80, *SD* = 12.44; 21 men, 97 women, 20 gender diverse/prefer not to report) and Chinese Australian (*n *= 129, *M*age = 30.16, *SD* = 8.93, 21 men, 97 women, 20 gender diverse/prefer not to report) trauma survivors completed measures of appraisals, cultural values, and PTSD symptoms.

**Results:** Findings showed that the Chinese Australian group was associated with greater Chinese cultural beliefs about adversity (i.e. emphasizing the value of adversity and people’s ability to overcome adversity) and fewer fatalism appraisals (i.e. appraising one's destiny as externally determined), which in turn were atemporally associated with fewer PTSD symptoms; these atemporal indirect associations were moderated by self-construal and holistic thinking. The Chinese Australian group also reported fewer secondary control appraisals (i.e. attempts to change aspects of the self and accept current circumstances), which were atemporally associated with greater PTSD symptoms. In contrast, the European Australian group was associated with fewer primary control appraisals (i.e. perceived ability to personally change or control a situation), which were atemporally associated with greater PTSD symptoms.

**Conclusion:** These findings highlight the importance of considering the influence of culture on appraisals in PTSD. However, it must be noted that causal relationships cannot be inferred from cross-sectional mediation analyses and thus, future longitudinal research is needed.

## HIGHLIGHTS

- Chinese Australian trauma survivors were associated with greater reporting of Chinese cultural beliefs about adversity and fewer fatalism appraisals, which were associated with fewer PTSD symptoms. These associations were moderated by a trauma survivor’s self-construal and level of holistic thinking.
- Chinese Australian trauma survivors reported fewer secondary control appraisals, which were associated with greater PTSD symptoms.
- European Australian trauma survivors were associated with fewer primary control appraisals, which were associated with greater PTSD symptoms.

## Abstract (es)

**Antecedentes:** Las evaluaciones son centrales en el trastorno de estrés postraumático (TEPT). Aun así, pocos estudios han examinado cómo la cultura influye en la asociación entre los diferentes tipos de evaluaciones relacionadas con el trauma y los síntomas de TEPT.

**Objetivo:** Este estudio investigó las influencias culturales en las evaluaciones del control y sus asociaciones con los síntomas de TEPT.

**Método:** Sobrevivientes de trauma europeos-australianos (*n** *= 140 edad *M** *= 35.80, *DE** *= 12.44, 21 hombres, 97 mujeres, 20 de género diverso/prefiere no reportar) y chino-australianos (*n** *= 129, Edad *M** *= 30.16, *DE** *= 8.93, 21 hombres, 97 mujeres, 20 de género diverso/prefiere no reportar) completaron medidas de evaluaciones, valores culturales y síntomas de TEPT.

**Resultados:** Los hallazgos mostraron que el grupo chino-australiano estaba asociado con mayores creencias culturales chinas acerca de la adversidad (es decir, énfasis en el valor de la adversidad y la capacidad de las personas para sobreponerse a la adversidad) y menos evaluaciones fatalistas (es decir, evaluación que el destino de uno está determinado externamente), que a su vez estuvieron asociados atemporalmente con menos síntomas de TEPT; estas asociaciones indirectas atemporales estuvieron moderadas por un pensamiento holístico y de autoconcepto. El grupo chino-australiano también reportó menos evaluaciones de control secundarias (es decir, intentos de cambiar aspectos de sí mismo y aceptar las circunstancias actuales), y que se asociaron atemporalmente con mayores síntomas de TEPT. En contraste, el grupo europeo-australiano se asoció con menos evaluaciones de control primario (es decir, capacidad percibida para cambiar o controlar personalmente una situación), las cuales se asociaron atemporalmente con mayores síntomas de TEPT.

**Conclusión:** Estos hallazgos resaltan la importancia de considerar la influencia de las evaluaciones culturales en el TEPT. Sin embargo, debe tenerse en cuenta que las relaciones causales no se pueden inferir a partir del análisis de mediación transversal y, por lo tanto, se necesitan futuras investigaciones longitudinales.

## 1. Introduction

Appraisals play a central role in the development, maintenance, and treatment of posttraumatic stress disorder (PTSD) (Bryant & Guthrie, 2005; Dunmore et al., 2001; Kleim et al., 2013). Maladaptive appraisals perpetuate a current sense of threat, initiate poor coping, and maintain PTSD symptoms (Ehlers & Clark, 2000). Decades of research have identified the types of appraisals associated with PTSD. A significant focus has been on the role of appraisals of a lack of perceived primary control (i.e. perceived ability to personally change or control a situation; Heckhausen & Schulz, 1995) in the context of posttraumatic psychological adjustment (Dunmore et al., 2001; Hancock & Bryant, 2020).

Cognitive models of PTSD, supported by significant empirical research, posit appraisals involving a lack of perceived control are important to development and maintenance of the disorder, as such appraisals perpetuate a sense of current threat and lead to maladaptive coping (e.g. Dunmore et al., 2001; Ehlers & Clark, 2000; Hancock & Bryant, 2018). Empirical literature has established that lower perceived control is associated with greater PTSD severity (Molnar et al., 2021) and PTSD has been characterized as a sense of loss of control (Hancock & Bryant, 2020). A sense of present and future control is associated with fewer PTSD symptoms, while appraisals relating to past control have been associated with greater distress (Frazier et al., 2001; Hancock & Bryant, 2018). Moreover, it has been highlighted that evidence-based treatment for PTSD should focus on assisting an individual recover a sense of control (Gómez de La Cuesta et al., 2019).

Despite the advances in understanding the role of appraisals in PTSD, there remains a concerning gap. This research has been predominantly conducted in Western cultural contexts (Bernardi et al., 2019). This is problematic because culture influences how individuals appraise everyday experiences (Mesquita & Walker, 2003). It follows that such cultural differences extend to the appraisals of trauma (Bernardi & Jobson, 2019). Several theories have conceptualized two key dimensions by which cultural groups systematically vary (a) self-construal, and (b) analytic versus holistic thinking style.

Self-construal refers to the way individuals perceive the self in relation to others (Markus & Kitayama, 2010). Members of individualistic (e.g. Western Europe, US, Australia) cultures tend to value an independent self-construal; the self is perceived to be unique and separate from others, and personal primary control (i.e. autonomy) is valued (Markus & Kitayama, 2010). In contrast, members of collectivistic cultures (e.g. Asian, Middle Eastern) tend to value an interdependent self-construal; the self is perceived to be interconnected with others and group harmony is valued, and individual and personal control are less emphasized (Markus & Kitayama, 2010).

Analytic versus holistic thinking style refers to how individuals understand causality and attend to relationships between objects and their context (Nisbett et al., 2001). Members of Western cultures tend to endorse an analytic thinking style, which is characterized by differentiating focal people or objects from their contexts and ascribing causality to people or objects (Koo et al., 2018). Members of Asian cultures tend to adopt a holistic thinking style, where attention is given to relationships between objects and their contexts and causality is attributed to contexts (Koo et al., 2018).

Cultural variations in these two key dimensions influence appraisals of control. Those from Western cultures tend to appraise success through a sense of personal primary control, given their focus on analytic thinking (i.e. ascribing causality to people) and personal control being fundamental to the independent self (Bernardi et al., 2019; Markus & Kitayama, 2010; Mesquita & Walker, 2003; Shek & Yu, 2014). In Asian cultures, primary control has less relevance, given the focus on holistic thinking and interdependence (Bernardi et al., 2019; Markus & Kitayama, 2010; Mesquita & Walker, 2003). While PTSD research, given its Western focus, has predominantly focused on primary control (Bernardi et al., 2019), secondary control (i.e. attempts to change some aspect of the self and accept current circumstances) is proposed to be of greater relevance for Asian cultures, as it refers to aligning with the social context and attributing causality to the context (Bernardi et al., 2019).

Conceptual frameworks highlight violations to cultural beliefs are associated with psychopathology (Hwang et al., 2008; Jobson, 2009; Wong et al., 2010). Specifically, beliefs and appraisals associated with psychopathology need to be considered within the socio-cultural context of an individual; it is not merely that certain appraisals are universally ‘adaptive’ or ‘maladaptive’, but rather when beliefs and appraisals misalign with cultural norms and expectations there is the potential for psychopathology (Hwang et al., 2008; Jobson, 2009; Wong et al., 2010). Therefore, a lack of perceived primary control is proposed to be more strongly associated with PTSD symptoms for those from Western cultures, while a lack of perceived secondary control is proposed to be more strongly associated with PTSD symptoms for those from Asian cultures. Emerging research supports these predictions (Bernardi & Jobson, 2019; Cheng et al., 2013; Engelbrecht & Jobson, 2014; Jobson et al., 2022a; Reyneke et al., 2023). Integrating the above cross-cultural frameworks with PTSD theories (Ehlers & Clark, 2000), it is posited that for Western trauma survivors a perceived lack of primary control is associated with greater PTSD symptomatology, with these associations being stronger for those valuing independence (Bernardi et al., 2019; Markus & Kitayama, 2010) and analytic thinking (Bernardi et al., 2019; Mesquita & Walker, 2003). In contrast, for Asian trauma survivors a perceived lack of secondary control is associated with greater PTSD symptomatology, with these associations being stronger for those valuing interdependence (Bernardi et al., 2019; Markus & Kitayama, 2010) and holistic thinking (Bernardi et al., 2019). However, to date, these predictions have not been examined.

When considering beliefs about control in a post-trauma context, appraisals of fatalism and Chinese cultural beliefs about adversity are posited to be of importance for Asian trauma survivors (Bernardi et al., 2019; Shek & Yu, 2014; Xie & Wong, 2021). Fatalism is a belief about external control of life events (Zuo et al., 2020) and refers to accepting the situation based on a belief that destiny is ruled by a form of higher power (Maercker et al., 2019). Fatalistic appraisals are associated with greater PTSD symptom severity, as they can be generated by the failure to prevent or cope with stressful life events and can trigger maladaptive outcomes (Maercker et al., 2019; Navarro et al., 2018).

Due to traditional Chinese philosophies of Confucianism, Buddhism and Taoism, Asian cultural groups are more accepting of fatalism than Western groups (Zuo et al., 2020). Thus, fatalistic appraisals may be less detrimental, and potentially even beneficial, on psychological outcomes for those from Asian backgrounds (Bernardi et al., 2019; Heiniger et al., 2015). Emerging research indicates that Malaysian trauma survivors reported significantly greater appraisals of fatalism than Australian trauma survivors (Jobson et al., 2022b) and the positive association between fatalism and PTSD symptoms was significantly larger for European Americans than Asian Americans (Jobson et al., 2022a). Thus, when integrating PTSD and cross-cultural theoretical accounts, it is posited that for Asian trauma survivors, greater endorsement of appraisals of fatalism is associated with less PTSD symptomatology, with these associations being stronger for those valuing interdependence (Bernardi et al., 2019; Markus & Kitayama, 2010) and holistic thinking (Bernardi et al., 2019).

Chinese culture also has specific beliefs and appraisals about adversity (Xie & Wong, 2021). These beliefs are shaped by Confucianism, Buddhism and Taoism, whereby people’s inner virtues and strengths are strongly maintained (Shek & Yu, 2014; Xie & Wong, 2021). Within Chinese culture, adversity is perceived as being character-building and strengthening of psychological well-being (Shek et al., 2003; Xie & Wong, 2021). There is value in the acceptance of adversity, rather than individuals actively controlling or changing adverse events (Shek et al., 2003; Xie & Wong, 2021). Moreover, responding to adversity is perceived as being relational rather than individual-focused (Shek et al., 2003; Xie & Wong, 2021). Shek and colleagues (2003) developed a measure (Chinese Cultural Beliefs about Adversity Scale) that assessed these cultural beliefs about adversity. Recent studies using this measure have highlighted these cultural beliefs about adversity were more prevalent among Asian trauma survivors (Malaysian, Asian American) than Western trauma survivors (European Australian, European American) (Jobson et al., 2022a, b). Again, when integrating PTSD and cross-cultural theoretical accounts, it is posited that for Asian trauma survivors, greater endorsement of these cultural beliefs about adversity may be associated with less PTSD symptomatology, with these associations being stronger for those valuing interdependence (Bernardi et al., 2019; Markus & Kitayama, 2010) and holistic thinking (Bernardi et al., 2019). Despite the proposed importance of appraisals of fatalism and Chinese cultural beliefs about adversity, very few studies have examined the role that these appraisal types have in influencing posttraumatic stress responses among Chinese trauma survivors.

### 1.1. Current study

**Figure 1.**
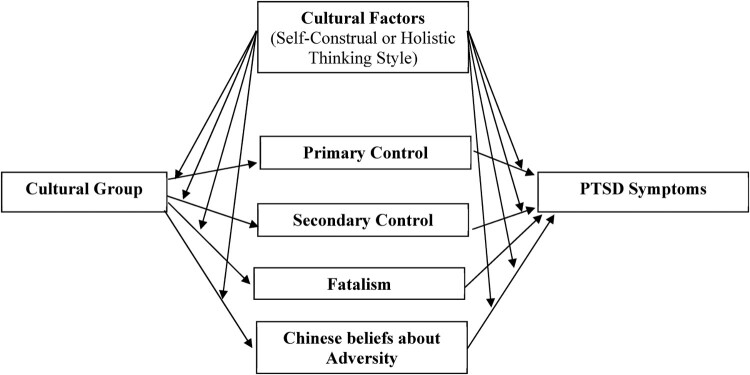
The proposed moderated mediation model for Hypothesis 3.

This study aimed to investigate the associations between appraisals of control and PTSD symptoms among Chinese^1^ trauma survivors residing in Australia (hereon referred to as the ‘Chinese Australian group’) and Australian with European heritage trauma survivors residing in Australia (hereon referred to as the ‘European Australian group’). Specifically, we aimed to examine (1) cultural group differences in control appraisals (i.e. primary, secondary, fatalism, Chinese cultural beliefs about adversity); (2) whether control appraisals mediated the correlation between cultural group and PTSD symptoms; and (3) whether the indirect association between cultural group and PTSD symptoms through appraisals was moderated by self-construal or holistic thinking style (moderated mediation model, Figure 1).

The mediation models specified in this study were grounded using a priori theoretical frameworks. Theoretical models of PTSD (Ehlers & Clark, 2000) and a large empirical body of research (Bryant & Guthrie, 2005; Dunmore et al., 2001; Kleim et al., 2013) have established causal relationships among the variables in our mediation model (i.e. a strong causal role of appraisals in PTSD) (Winer et al., 2016). However, it is important to note that our mediation aims and hypotheses were atemporal and thus, causality cannot be inferred from the statistical analyses (Winer et al., 2016). Rather the study is important as an initial study, given the concerning lack of cross-cultural studies examining trauma-related appraisals, in that it explores potential mediation pathways, and comparative analyses across these two cultural groups, which can inform future research.

We predicted the Chinese Australian group would appraise trauma in relation to how they adapted to and accepted current circumstances, and how they employed cultural belief systems that served as sources of strength when facing adversity. In contrast, the European Australian group would be more likely to appraise trauma in relation to perceived personal control. We hypothesized the Chinese Australian group would score higher when reporting appraisals of Chinese cultural beliefs about adversity, fatalism, and secondary control and lower when reporting appraisals of primary control compared to the European Australian group (Hypothesis 1). Second, we predicted the relationship with PTSD symptoms would be, for the Chinese Australian group, indirectly associated via less endorsement of Chinese cultural beliefs about adversity, fatalism, and secondary control, while the European Australian group would be indirectly associated through less primary control (Hypothesis 2). Finally, we predicted self-construal and holistic thinking style would moderate the atemporal indirect associations specified in Hypotheses 2. Specifically, we hypothesized that stronger interdependent self-construal (Hypothesis 3a) and stronger holistic thinking (Hypothesis 3b), would strengthen the mediation effects of Chinese cultural beliefs about adversity, fatalism and secondary control and weaken the mediation effects of primary control.

## 2. Method

### 2.1. Design

To examine our aims, we used a cross-sectional survey design with two cultural groups: an Australian with European heritage group and a Chinese group residing in Australia for less than 15 years. The 15-year timeframe was decided for two primary reasons. First, after consulting Chinese researchers and community members, 15 years and less was deemed an appropriate time to capture the inherent Chinese culture embedded by this group of participants, while minimizing the potential influences of long-term residency on cultural identity, cognitions and behaviours. Second, we selected 15 years to account for the special circumstances concerning COVID-19, in which Australia closed its borders to China for three years. Thus, if we had made the time of residing in Australia too short, we would have mainly captured international students who had recently arrived after the borders reopened, rather than a diverse population that represents the Chinese community in Australia.^2^ Data for this study can be accessed at https://osf.io/bj6gd/. The study protocol complies with the local ethical regulations and the Declaration of Helsinki and was approved by the Monash University Human Research Ethics Committee (32521).

### 2.2. Participants

Participants were recruited from the general community in Australia via online social media advertisements on Facebook, WeChat, and RED. Inclusion criteria were (a) aged between 18 and 65 years, (b) experienced a Criterion A traumatic event, (c) were either born in Australia and culturally self-identified as Australian with European heritage OR born in China, and culturally self-identified as Chinese and moved to Australia within the last 15 years, (d) all four grandparents were of the same cultural background as the participant, and (e) able to complete the survey in either English or simplified Chinese.

Participants were screened against inclusion criteria via email following registration of interest. Eligible participants were then emailed a link to the online survey. Those who completed the survey received a $25AUD gift card. A total of 388 survey responses were collected and subsequently assessed for quality. Responses were excluded if (a) the index trauma did not meet Criterion A (in accordance to the Life Event Checklist-5); (b) the reCAPTCHA score (generated by Qualtrics) was below 0.5, which indicates a non-human, machine-automatic response; (c) responses were recorded at latitude or longitude levels outside Australia; (d) the Conscientious Responder Scale score was ≤ 2 (Marjanovic et al., 2014), which indicates an unconscientious response; or (e) participants completed the survey in under 15 min (Jobson et al., 2022b). Based on these criteria, 119 responses were excluded.

The final sample included 269 (European Australian *n *= 140, Chinese *n *= 129) trauma survivors. The mean age of the *European Australian* group was 35.80 years (*SD* = 12.44), and the mean age of the Chinese Australian group was 30.16 years (*SD* = 8.93). The European Australian group consisted of 21 men, 97 women and 20 gender diverse individuals/participants who preferred not to report their gender. The Chinese Australian group consisted of 31 men, 95 women and 2 gender-diverse individuals/ participants who preferred not to report their gender. This sample size exceeded the a priori power analysis, whereby for Hypothesis 1, using G Power (with a moderate effect size; Bernardi & Jobson, 2019, alpha = .05 and 80% power), we found 196 participants (98 per cultural group) were needed. Using the approach suggested by Schoemann et al. (2017), we found that a sample size greater than 250 would provide adequate power for each of the indirect effects tested in Hypothesis 2. Additionally, our sample size exceeded the minimum sample size suggested for research using moderated mediation (Chu & Chen, 2012).

### 2.3. Measures

#### 2.3.1. Life Events Checklist-5 (LEC-5)

The LEC-5 assessed participants’ lifetime exposure to Criterion A traumatic events (Weathers et al., 2013). Participants self-reported their exposure to 16 types of traumatic events on 6-point scales (happened to me, witnessed it, learned about it, part of my job, not sure, doesn’t apply). Example events included natural disaster, physical assault, and severe human suffering. Afterwards, participants nominated their index traumatic event (i.e. the trauma that most affected them) and the time since their index trauma (Weathers et al., 2013). Based on our inclusion criteria, individuals who did not endorse experiencing any traumatic events, based on responses from the LEC-5, were automatically screened out from the survey. Participants reported a range of index trauma types, which were subsequently coded into accidents, non-sexual assaults, sexual assaults, unexpected death, natural disasters, and others (See Table 1).

**Table 1.** Summary of Group Characteristics.

Variable	Chinese Australian Group (*n *= 129)	European Australian Group (*n *= 140)	Statistics
Age - years *M* (*SD*)	30.16 (8.93)	35.80 (12.44)	*F*(1,264) = 17.81** η_p_^2 ^= .06
Gender (*n*)			
men:women:gender diverse/prefer not to report	31:95:2	21:97:20	X^2^(*df *= 2, *N *= 266) = 16.32**
Education (*n*) Secondary: Post-Secondary: Degree: Postgraduate degree: Other: Prefer not say	13:8:42:65:0:0	20:19:48:46:4:1	X^2^(*df *= 4, *N *= 266) = 14.26*,
Trauma type (*n*)			
Accident: Non-Sexual Assault: Sexual Assault: Unexpected death: Natural disaster: Other	22:47:9:31:16:4	16:41:34:42:2:5	X^2^(*df *= 5, *N *= 269) = 28.15**
Time since trauma – years *M* (*SD*)	8.08 (7.50)	13.11 (11.73)	*F*(1,266) = 17.11** η_p_^2 ^= .06
PTSD symptoms *M* (*SD*)	27.19 (16.86)	35.23 (18.56)	*F*(1,267) = 13.76** η_p_^2 ^= .05
Self-construal ratio (independent-interdependent) *M* (*SD*)	−0.77 (14.01)	5.26 (21.10)	*F*(1,266) = 7.43* η_p_^2 ^= .03
Holism Total *M* (*SD*)	76.64 (9.21)	74.21 (9.04)	*F*(1,259) = 70.56** η_p_^2 ^= .21
**Control Variables**			
Primary Control *M* (*SD*)	44.95 (10.18)	34.51 (9.94)	*F*(1,259) = 70.56** η_p_^2 ^= .21
Secondary Control *M* (*SD*)	55.05 (9.48)	47.93 (8.03)	*F*(1,259) = 48.15** η_p_^2 ^= .16
Fatalism *M* (*SD*)	3.23 (.86)	2.70 (.86)	*F*(1, 258) = 23.37 ** η_p_^2 ^= .08
Chinese cultural beliefs about adversity *M* (*SD*)	33.79 (5.87)	28.09 (6.28)	*F*(1,258) = 42.42** η_p_^2 ^= .14

Note: *p < .01, **p < .001.

#### 2.3.2. PTSD Checklist for the DSM-5 (PCL-5)

The PCL-5 is a 20-item self-report measure of PTSD symptom severity (Weathers et al., 2013). Participants completed the PCL-5 with respect to their index trauma (as indicated in the LEC-5) and rated how much they were bothered by each symptom in the past month on 5-point Likert scales (0 = *not at all*, 4 = *extremely*), with higher scores indicating greater symptom severity. The PCL-5 has good psychometric properties in cross-cultural research (Cheng et al., 2020). In this study, the PCL-5 showed excellent internal consistency for both the European Australian group (Cronbach’s alpha = .94) and Chinese Australian group (Cronbach’s alpha = .94).

#### 2.3.3. Primary-Secondary Control Scale (PSCS)

The PSCS is a 37-item self-report questionnaire measuring appraisals of primary control (17 items) and secondary control (20 items) (Chang et al., 1997). Participants rated their agreement with each item on 4-point Likert scales (1 = *totally disagree*, 4 = *totally agree*) while thinking about their index trauma. Scores were summed for primary and secondary subscales. The PSCS has been used in previous cross-cultural research (Reyneke et al., 2023). In this study, the PSCS showed good internal consistency for the European Australian group (primary control Cronbach’s alpha = .89; secondary control Cronbach’s alpha = .76) and Chinese Australian group (primary control Cronbach’s alpha = .91; secondary control Cronbach’s alpha = .85).

#### 2.3.4. Fatalism Questionnaire

The Fatalism Questionnaire consists of six items measuring participants’ beliefs in how much of their life is determined by external factors outside of one’s control (Maercker et al., 2019). Participants rated their agreement with each item on 5-point Likert scales (1 = *strongly disagree*, 5 = *strongly agree*). Mean scores were calculated, with higher scores reflecting greater fatalistic beliefs. This questionnaire has good psychometric properties in cross-cultural research (Maercker et al., 2019). In this study, internal consistency was good for the European Australian group (Cronbach’s alpha = .81) and Chinese Australian group (Cronbach’s alpha = .83).

#### 2.3.5. Chinese Cultural Beliefs about Adversity Scale (C-CBAS)

The C-CBAS is a 9-item self-report measure of Chinese cultural beliefs about adversity (Shek et al., 2003). It contains seven items that highlight positive appraisals about adversity (i.e. cultural beliefs emphasising the positive value of adversity and people’s capacity to overcome adversity and two reversed scored items that highlight negative beliefs about adversity). Participants rated their agreement with each item on 6-point Likert scales (1 = *totally disagree* to 6 = *totally agree*), with higher scores reflecting greater positive cultural beliefs of adversity. In this study, the CBAS demonstrated acceptable internal consistency for the European Australian group (Cronbach’s alpha = .70) and Chinese Australian group (Cronbach’s alpha = .73).

#### 2.3.6. Holistic Cognitions Scale (HCS)

The HCS is a 16-item self-report measure of different cultural thinking styles, namely holistic and analytic thinking (Lux et al., 2021). Items are divided into four constructs (four items each) that differ between holistic and analytic thinking: attention, causality (reverse scored), contradiction, and perception of change (reverse scored). Participants rated their agreement with each item on 7-point Likert scales (1 = *completely disagree*, 7 = *completely agre*e). Higher total scores indicated greater holistic thinking. In this study, the HCS showed adequate internal consistency for the European Australian group (Cronbach’s alpha = .60) and Chinese Australian group (Cronbach’s alpha = .69).

#### 2.3.7. Self-Construal Scale (SCS)

The SCS is a 30-item self-report measure of how people define themselves in relation to others (Kitayama et al., 2014). It consists of two subscales: independent (15 items) and interdependent (15 items). Participants rated their agreement with each statement on 7-point Likert scales (1  = *strongly disagree*, 7 = s*trongly agree*) and scores were summed for both subscales. Following the approach of past research (Chiao et al., 2009; Liddell et al., 2015, 2017), we computed a self-construal index by subtracting total interdependent self from total independent self. The SCS showed good internal consistency for independent and interdependent subscales (European Australian group – independent Cronbach’s alpha = .81, interdependent Cronbach’s alpha = .80; Chinese Australian group – independent Cronbach’s alpha = .76, interdependent Cronbach’s alpha = .78).

#### 2.3.8. Conscientious Responder Scale (CRS)

The CRS is a validity check (Marjanovic et al., 2014) consisting of five items that instruct participants to respond in a specific way (e.g. To answer this question, please choose option number four, ‘neither agree nor disagree’). Each item was randomly placed within the survey to discern attentive/conscientious responding from inattentive/non-conscientious responding. Correct responses were scored with a 1 and incorrect responses were scored with a 0. Scores were summed and participants with CRS scores ≤ 2 were excluded from data analysis (Marjanovic et al., 2014).

### 2.4. Procedure

Eligible participants were invited to complete an online survey on Qualtrics. Participants provided informed consent. The questionnaires were presented in English for the European Australian group and in simplified Chinese for the Chinese Australian group. All measures not available in simplified Chinese were back-translated using gold-standard procedures (Gorecki et al., 2014).

### 2.5. Data analysis plan

Data was analysed using SPSS v28. Prior to hypothesis testing, we conducted measure invariance testing using R. We ran multiple multigroup (Chinese Australian vs European Australian) confirmatory factor analyses for each measure. All model fits were guided by Hu and Bentler’s (1999) guidelines for good model fits. Based on results from configural, metric and scalar models, we were confident that our measures indexed similar constructs across both cultural groups. As noted below, there were several group differences. Thus, we included age, gender, education, index trauma type, and time since trauma as covariates in all analyses.^3^

To assess Hypothesis 1, we used a series of analysis of covariances (ANCOVAs) to compare the two cultural groups on the appraisals investigated in this study. To adjust for multiple comparisons, we applied Bonferroni adjustment; α<.0125. To assess Hypothesis 2, we conducted a parallel mediation analysis using PROCESS (Model 4; Hayes, 2017). The model assessed whether control appraisals (Chinese cultural beliefs about adversity, fatalism, secondary control, primary control) mediated the relationship between cultural group and PTSD symptoms (Hypothesis 2). In the mediation analyses, the independent variable was cultural group (used as a binary variable, with European Australian group being coded as 1 and Chinese Australian group coded as 2), the dependent variable was PTSD symptom severity, and the potential mediator variables were the appraisals. Significance was indicated by the 95% confidence intervals not including 0.

To assess Hypothesis 3, we conducted two moderating mediation analyses using PROCESS (Model 58; Hayes, 2017). Two moderating mediation models examined whether level of self-construal (Hypothesis 3a) and holistic thinking style (Hypothesis 3b) moderated any mediating effects of control appraisals on the relationship between cultural group and PTSD symptoms. As the self-construal index (i.e. independent minus interdependent self-construal) was a continuous variable, the moderating effects of self-construal index were considered at low self-construal (1SD below the mean), medium self-construal (mean) and high self-construal (1SD above the mean). As the mean for the self-construal index was close to zero (*M *= 2.38, *SD *= 18.28), (a) low self-construal index indicated relatively stronger preference for interdependent self-construal, (b) medium self-construal indicated no strong preference for either independent or interdependent self-construal, and (c) high self-construal index indicated relatively stronger preference for independent self-construal.

## 3. Results

### 3.1. Participant characteristics

Participant characteristics are presented in Table 1 (see Supplementary Table 1 for correlation analyses between study variables for each cultural group). The groups differed significantly in age (European Australian group was older), gender (European Australian group had more gender diverse/prefer not to report gender participants), education status (see Table 1 for distribution), time since trauma (Chinese Australian group had a more recent index trauma), and index trauma type. The most reported index trauma type for the Chinese Australian group was non-sexual assault, while for the European Australian group it was unexpected death. The European Australian group reported notably more sexual assaults than the Chinese Australian group (see Table 1 for further details).

As expected, the European Australian group reported significantly greater independent self-construal relative to interdependent self-construal than the Chinese Australian group. The Chinese Australian group also reported significantly greater holistic thinking style than the European Australian group. The European Australian group scored significantly higher on PTSD symptom severity. Given these cultural group differences in PTSD symptoms, we also created a secondary dataset based on case matching (using SPSS v 28). European Australian participants were case matched to Chinese Australian participants based on their PCL-5 total scores, using a match tolerance of 5. This led to 113 matches and 27 participants did not have a match and were excluded. Thus, the secondary data set included 226 participants (113 per cultural group). We used this secondary dataset to also examine each of the hypotheses using the same analyses as that reported in the Data Analysis Plan section. For each hypothesis, the same pattern of results emerged to that reported below.

### 3.2. Hypothesis 1: cultural group differences in appraisals

As shown in Table 1, in support of Hypothesis 1, the Chinese Australian group endorsed significantly greater Chinese cultural beliefs about adversity, fatalism, and secondary control appraisals than the European Australian group. However, contrary to Hypothesis 1, the Chinese Australian group also scored significantly higher on primary control appraisals.

### 3.3. Hypothesis 2: appraisals as atemporal mediators of the association between cultural group and PTSD symptoms

There was an indirect effect from cultural group to PTSD symptoms through Chinese cultural beliefs about adversity, B = −2.40, *SE *= 1.03, 95%CI −4.57, −0.57, fatalism, B = 2.40, *SE *= .82, 95%CI 0.91, 4.12, secondary control, B = −6.07, *SE *= 1.44, 95%CI −9.10, −3.47, and primary control, B = 6.74, *SE *= 1.56, 95%CI 3.98, 10.16. Specifically, in support of Hypothesis 2, the Chinese Australian group was associated with fewer appraisals of Chinese cultural beliefs about adversity and secondary control, which were in turn atemporally associated with higher PTSD symptoms. The European Australian group was associated with fewer appraisals of primary control, which was in turn atemporally associated with higher PTSD symptoms. Contrary to Hypothesis 2, the Chinese Australian group was associated with greater appraisals of fatalism, which was in turn atemporally associated with higher PTSD symptoms.

### 3.4. Hypothesis 3a: control appraisals as atemporal mediators and self-Construal as moderator

Statistical results for Hypothesis 3a are presented in Table 2. In this section we consider whether self-construal moderated the atemporal mediation effects outlined above in Hypothesis 2, as illustrated in Figure 1. The atemporal mediation effect of cultural group being associated with PTSD symptoms through Chinese cultural beliefs about adversity was significantly moderated by self-construal; significant for low self-construal index, B = −4.79, *SE *= 1.96, 95%CI −9.25, −1.56, and medium self-construal index, B = −2.48, *SE *= 1.08, 95%CI −4.86, −0.70, but not high self-construal index, B = −0.57, *SE *= 1.16, 95%CI −3.06, 1.60. The atemporal mediation effect of cultural group being associated with PTSD symptoms through fatalism was moderated by self-construal index; significant for those with medium self-construal index, B = 2.19, *SE *= 0.83, 95%CI 0.71, 3.98, and high self-construal index, B = 2.94, *SE *= 1.44, 95%CI 0.66, 6.19, but not low self-construal index, B = 1.51, *SE *= 0.98, 95%CI −0.07, 3.74. The atemporal mediation effect of cultural group being associated with PTSD symptoms through primary control was significant regardless of self-construal level; low self-construal index, B = 5.07, *SE *= 1.96, 95%CI 1.59, 9.36, medium self-construal index, B = 7.13, *SE *= 1.62, 95%CI 4.23, 10.52, and high self-construal index, B = 9.53, *SE *= 2.67, 95%CI 5.02, 15.26. Similarly, the atemporal mediation effect of cultural group being associated with PTSD symptoms through secondary control was significant regardless of self-construal level; low self-construal index, B = −3.53, *SE *= 1.61, 95%CI −7.06, −0.80, medium self-construal index, B = −6.55, *SE *= 1.53, 95%CI −9.76, −3.80, and high self-construal index, B = −10.46, *SE *= 2.74, 95%CI −16.42, −5.56. The findings related to the other significant pathways in this model are presented as Supplemental Material.

**Table 2.** Moderated mediation analyses for self-construal on the association between cultural group and PTSD symptoms through control appraisals

	B	*SE*	*t*	*p*	95%CI
Outcome Variable: Chinese Cultural Beliefs about adversity					
Cultural group	5.37	.85	6.34	<.01	3.71-7.05
Self-Construal	-.004	.02	.19	.85	-.05,.04
Cultural group x Self-Construal	-.03	.05	.70	.48	-.12,.06
Outcome Variable: Fatalism					
Cultural group	.53	.12	4.47	<.01	.30,.76
Self-Construal	-.01	.003	2.86	<.01	-.02,-.003
Cultural group x Self-Construal	.01	.01	1.22	.22	-.005,.02
Outcome Variable: Primary Control					
Cultural group	11.85	1.39	8.53	<.01	9.11,14.59
Self-Construal	.10	.04	2.63	<.01	.03,.17
Cultural group x Self-Construal	.09	.08	1.24	.22	-.05,.24
Outcome Variable: Secondary Control					
Cultural group	8.41	1.20	6.99	<.01	6.04,10.78
Self-Construal	.07	.03	2.03	.04	.002,.13
Cultural group x Self-Construal	.10	.07	1.57	.12	-.03,.23
Outcome Variable: PTSD Symptoms					
Chinese Cultural Beliefs about Adversity	-.46	.17	2.67	<.01	-.80,-.12
Fatalism	4.14	1.16	3.56	<.01	1.85,6.43
Primary Control	.60	.11	5.58	<.01	.39,.81
Secondary Control	-.78	.13	5.85	<.01	−1.04,-.52
Chinese Cultural Beliefs about Adversity x Self-Construal	.02	.01	1.99	.05	.002,.04
Fatalism x Self-Construal	.01	.06	.21	.83	-.10,.13
Primary Control x Self-Construal	.01	.01	.89	.38	-.01,.02
Secondary Control x Self-Construal	-.01	.01	1.95	.05	-.03,.0001

Note: Cultural group; 1 = European Australian group, 2 = Chinese Australian group.

### 3.5. Hypothesis 3b: control appraisals as atemporal mediators and holistic thinking as moderator

Table 3 presents the results for Hypothesis 3b. Here we consider whether holistic thinking moderated the atemporal mediation effects outlined above in Hypothesis 2. The atemporal mediation effect of cultural group being associated with PTSD symptoms through Chinese cultural beliefs about adversity was moderated by holistic thinking style; significant for those with medium holism, B = −2.61, *SE *= 1.06, 95%CI −4.86, −0.68, and high holism, B = −3.62, *SE* 1.74, 95%CI −7.16, −0.30, but not low holism, B = −1.64, *SE *= 1.26, 95%CI −4.56, 0.44. Similarly, the atemporal mediation effect of cultural group being associated with PTSD symptoms through fatalism was moderated by holistic thinking; significant for those with medium holism, B = 2.18, *SE *= .80, 95%CI .71, 3.86, and high holism, B = 2.21, *SE *= 1.02, 95%CI .50, 4.48, but not low holism, B = 1.94, *SE *= 1.47, 95%CI -.67, 5.06. The atemporal mediation effect of cultural group being associated with PTSD symptoms through primary control was significant for low holism, B = 5.93, *SE *= 2.16, 95%CI 2.55, 10.96, medium holism, B = 6.60, *SE *= 1.63, 95%CI 3.67, 10.10, and high holism, B = 7.30, *SE *= 2.37, 95%CI 2.67, 12.14. The atemporal mediation effect of cultural group being associated with PTSD symptoms through secondary control was significant for those with low holism, B = −5.65, *SE *= 1.62, 95%CI −9.09, −2.74, medium holism, B = −5.61, *SE *= 1.43, 95%CI −8.64, −3.10, and high holism, B = −5.56, *SE *= 2.04, 95%CI −10.05, −2.15.

**Table 3.** Moderated mediation analyses for holistic thinking style as a moderator of the association between cultural group and PTSD symptoms through control appraisals.

	B	*SE*	*t*	*p*	95%CI
Outcome Variable: Chinese Cultural Beliefs about adversity					
Cultural group	4.92	.80	6.18	<.001	3.35,6.49
Holism	.22	.04	5.54	<.001	.14,.29
Cultural group x Holism	.01	.08	.18	.86	-.14,.17
Outcome Variable: Fatalism					
Cultural group	.57	.12	4.72	<.001	.33,.80
Holism	.01	.006	1.74	.08	-.001,.03
Cultural group x Holism	-.01	.01	.98	.33	-.03,.01
Outcome Variable: Primary Control					
Cultural group	11.03	1.39	7.91	<.001	8.28,13.78
Holism	.07	.07	1.09	.28	-.06,.21
Cultural group x Holism	.08	.14	.62	.54	-.19,.35
Outcome Variable: Secondary Control					
Cultural group	7.74	1.20	6.47	<.001	5.38,10.09
Holism	.12	.06	2.21	.05	.003,.23
Cultural group x Holism	-.01	.12	.10	.92	-.24,.22
Outcome Variable: PTSD Symptoms					
Chinese Cultural Beliefs about Adversity	-.53	.19	2.86	<.01	-.90,-.16
Fatalism	3.85	1.15	3.35	<.01	1.58,6.11
Primary Control	.60	.11	5.53	<.001	.39,.81
Secondary Control	-.72	.14	5.30	<.001	-.99,-.46
Chinese Cultural Beliefs about Adversity x Holism	-.02	.02	1.13	.26	-.06,.02
Fatalism x Self-Holism	.10	.14	.73	.47	-.18,.39
Primary Control x Holism	.002	.01	.20	.84	-.02,.02
Secondary Control x Holism	-.001	.01	.04	.97	-.03,.03

Note: Cultural group; 1 = European Australian group, 2 = Chinese Australian group.

## 4. General discussion

This study aimed to investigate the associations between control appraisals and PTSD symptoms among European Australian and Chinese Australian trauma survivors. Chinese Australian trauma survivors reported significantly greater endorsement of appraisals emphasizing the (a) value of adversity (Chinese cultural beliefs about adversity), (b) acceptance of adversity (fatalism), and (c) adapting oneself in response to adversity (secondary control) than the European Australian group (Hypothesis 1). This extends past research by establishing the relevance of these appraisal types for Chinese trauma survivors (Bernardi et al., 2019; Jobson et al., 2022b; Shek et al., 2003; Xie & Wong, 2021). Regarding Hypotheses 2 and 3, while appraisals of fatalism and cultural beliefs emphasizing the value of adversity atemporally mediated the relationships between cultural group and PTSD symptoms, self-construal and holistic thinking moderated these pathways in specific ways. Additionally, the Chinese Australian group was associated with fewer appraisals of secondary control and the European Australian group was associated with fewer appraisals of primary control, which were both atemporally associated with greater PTSD symptom severity. There were no moderating effects of self-construal or holism on these associations.

The Chinese Australian group was associated with greater cultural beliefs about the value of adversity, which was associated with fewer PTSD symptoms. However, this was only observed for those valuing interdependence and holistic thinking style. This aligns with cultural theories positing the importance of self-construal and holistic thinking in shaping appraisals among those from Asian cultures (Bernardi et al., 2019; De Vaus et al., 2018). Chinese cultural beliefs emphasize the positive value of adversity and people’s capacity to overcome adversity (Shek & Yu, 2014; Xie & Wong, 2021). These beliefs are shaped by Confucian thoughts and influenced by Buddhism and Taoism (Shek & Yu, 2014). If longitudinal research replicates these findings, these characteristics may be protective for Chinese trauma survivors tending towards interdependence and holistic thinking (Xie & Wong, 2021).

Contrary to our hypothesis, the Chinese Australian group was associated with greater appraisals of fatalism, which was associated with greater PTSD symptomatology. Thus, regardless of cultural background, fatalism may be associated with greater PTSD symptomatology, as fatalistic appraisals can be generated by the failure to prevent or cope with stressful life events and can trigger maladaptive outcomes (Maercker et al., 2019; Navarro et al., 2018). Additionally, other studies have found that pan-culturally fatalism appraisals were associated with PTSD (Maercker et al., 2019). However, this atemporal mediation effect was only observed for those valuing independence and holistic thinking. Fatalism is the propensity of individuals to believe ‘their destinies are ruled by an unseen power or are played out inevitably rather than by their will’ (Maercker et al., 2019, p. 2). The indirect associations only being observed among those with greater independence may align with those with greater independence valuing personal mastery and thus, fatalistic appraisals may pose greater challenges to the independent self and consequently be associated with poorer mental health. The indirect association was also observed among those with greater holistic thinking, which may align with holistic thinking emphasizing causality being attributed to contexts (Koo et al., 2018).

The European Australian group was associated with fewer appraisals of primary control, which was associated with greater PTSD symptoms, indicating trauma survivors who appraised they could not personally change their current situation were experiencing greater PTSD symptoms. This pattern reflects the findings reported in the majority of PTSD research which has focused on primary control, possibly because this research has largely recruited Western-based samples. Uniquely, we found that the Chinese Australian group was atemporally associated with higher PTSD symptoms via fewer appraisals of secondary control. This suggests that the perceived limited ability to change some aspects of the self and accept the current circumstances may be an important culturally relevant appraisal to consider for posttraumatic recovery for Chinese trauma survivors. Interestingly, for both appraisals of primary and secondary control, these findings were evident regardless of levels of holistic thinking or self-construal, demonstrating the potential importance of these appraisals to distinguish cultural groups.

Evidence-based interventions for PTSD target trauma-related maladaptive appraisals (Ehlers & Clark, 2000). Significant research indicates the importance of cultural tailoring interventions, which improves treatment outcome (Huey Jr et al., 2014). Yet, while appraisals remain a significant target of PTSD interventions, little research is available to guide this cultural tailoring. The current study highlights that clinicians need to potentially consider Chinese cultural beliefs about adversity, fatalism, and secondary control when working with clients with Chinese backgrounds. However, it is important to highlight that the study indicated that these effects do not pertain merely to ethnicity but rather need to be considered in relation to a client’s thinking style and self-construal. Therefore, clinicians need greater consideration of the role of cultural factors, such as self-construal and analytic versus holistic thinking style. For instance, clinicians need to consider (a) how trauma impacts interdependent aspects of self and relational orientations, (b) that cultural identities, practices, and values provide meaning and strength following trauma, and (c) some cultural values emphasize accepting trauma and enduring suffering rather than trying to change aspects of it (see Xie & Wang, 2021). There is a need for further research and replication to further develop these clinical implications, but what is emerging is that it seems critical for clinicians to understand broader cultural differences in client experiences of trauma and PTSD and not assume the same pan-cultural negative/positive impacts of certain appraisals; despite decades of Western research focusing on these appraisal types.

There are several limitations. First, the study was cross-sectional, and the mediation models were atemporal. Hence, causality cannot be inferred. Rather the study provides explorations of potential mediation pathways, and comparative analyses across two cultural groups, and thus can inform the design of future longitudinal research. Second, our study did not examine a clinical sample albeit over 30% in each cultural group met provisional diagnosis for PTSD. Additionally, the European Australian group had significantly higher PTSD symptoms scores than the Chinese Australian group. Nonetheless, as noted, a similar pattern of results was found when we used a secondary case matched dataset. Third, we did not assess the extent to which participants subscribe to Confucianism/Taoist/Buddhist ideology, which would benefit future studies. Fourth, we acknowledge that China is a large and diverse country. Thus, the regions in which participants had migrated from may have influenced findings and therefore be considered in future studies. Finally, our two cultural groups differed on several demographic and trauma-related factors. While these factors were included as covariates in our analyses, these factors may have influenced findings, such as the intersectionality between culture, gender, and trauma type.

## 5. Conclusion

This was the first study to examine trauma-related appraisals among Chinese trauma survivors residing in Australia. Findings demonstrate cultural differences in the atemporal associations between appraisals relating to control and PTSD symptoms. Moreover, these associations were influenced by self-construal and analytic versus holistic thinking style. Overall, this study supports the idea that cultural factors influence how appraisals are associated with PTSD symptom severity. Given appraisals are targeted in evidence-based interventions for PTSD, further research regarding cultural influences on appraisals is needed to guide the cultural tailoring of these interventions.

## Supplementary Material

R1Supplemental Material_APPRAISAL PAPER.docx

## Data Availability

Data for this study can be accessed at https://osf.io/bj6gd/

## Notes

1 Chinese participants were from mainland China, Hong Kong and Macau. Participants could choose to complete the study in either English or simplified Chinese. Some participants in the Chinese group chose to complete the study in English.

2 One of the key inclusion criteria was that participants needed to self-identify as having Chinese cultural background. During recruitment, we closely monitored the cultural aspects of our inclusion criteria. Our sample data did not contain anyone who moved to Australia at a young age (but would still meet the criteria of having lived in Australia for less than 15 years). In times where participants may have suspected higher acculturation when compared to the rest of the sample due to their age and time lived in Australia, the research team discussed their eligibility in weekly meetings.

3 A similar pattern of results emerged when these variables were not included as covariates.
