# Supplementary material for: Cultural differences in appraisals of control and posttraumatic stress disorder symptoms
Source: Eur J Psychotraumatol. 2024 Jun 5;15(1):2358685. doi: 10.1080/20008066.2024.2358685 (PMC11155424; doi:10.1080/20008066.2024.2358685)
Supplement: R1Supplemental Material_APPRAISAL PAPER.docx [file ZEPT_A_2358685_SM5626.docx]

**Supplemental Material**

**Table S1**

*Correlations between study variables (primary control, secondary control, fatalism, Chinese cultural beliefs about adversity, self-construal, holistic thinking) for each cultural group*

| Variables | Primary Control | Secondary Control | Fatalism | Chinese Cultural Beliefs about Adversity | Self-construal Index | Holistic Thinking |
| --- | --- | --- | --- | --- | --- | --- |
| Primary Control | - | .32^**^ | .10 | .16^*^ | .12 | .01 |
| Secondary Control | .55^**^ | - | .19^*^ | .35^**^ | .07 | .11 |
| Fatalism | .01 | .11 | - | .12 | -.30^**^ | .17^*^ |
| Chinese Cultural Beliefs about Adversity | .16 | .33^**^ | -.12 | - | .06 | .33^**^ |
| Self-construal Index | .20^*^ | .18^*^ | -.08 | -.034 | - | .03 |
| Holistic Thinking | .12 | .12 | .05 | .36^**^ | .11 | - |

*Note.* Correlations between study variables for the European Australian group were reported above the diagonal, correlations between study variables for the European Australian group were reported above the diagonal for the Chinese Australian group was reported below the diagonal. Self-construal index was calculated by subtracting total interdependent self from total independent self. **Correlations is significant at the .01 level (2-tailed), *Correlation is significant at the .05 level (2-tailed).

**Hypothesis 3 - Additional Significant Pathways**

**Additional Significant Pathways for Control Appraisals as Mediators and Self-Construal as Moderator**

Regarding the other significant pathways in this model (see Table 2), self-construal moderated the association between greater appraisals of Chinese cultural beliefs about adversity and fewer PTSD symptoms, whereby the negative association was significant for low self-construal index, B= -0.80, *SE*= 0.26, *t*= 3.13, *p*<.01, 95% CI -1.31, -0.30, and medium self-construal index, B= -0.46, *SE*= 0.17, *t*= 2.67, *p*<.01, 95% CI -0.80, -0.12, but not high self-construal index, B= -0.12, *SE*= .23, *t*= 0.52, *p*=.60, 95% CI -0.57, 0.33 (Supplemental Figure 1). Self-construal also moderated the association between fewer appraisals of secondary control and greater PTSD symptoms regardless of level of self-construal; low self-construal, B= -0.54, *SE*= 0.17, *t*= 3.09, *p*<.01, 95%CI -0.89, -0.20, medium self-construal, B= -0.78, *SE*= 0.13, *t*= 5.85, *p*<.001, 95%CI -1.04, -0.52, high self-construal, B= -1.02, *SE*= 0.19, *t*= 5.47, *p*<.001, 95%CI -1.38, -0.65 (Supplemental Figure 2).

**Supplemental Figure 1**

*Moderating effects of Self-Construal (Independent relative to Interdependent) on Chinese Cultural Beliefs about Adversity and PTSD Symptoms*

**
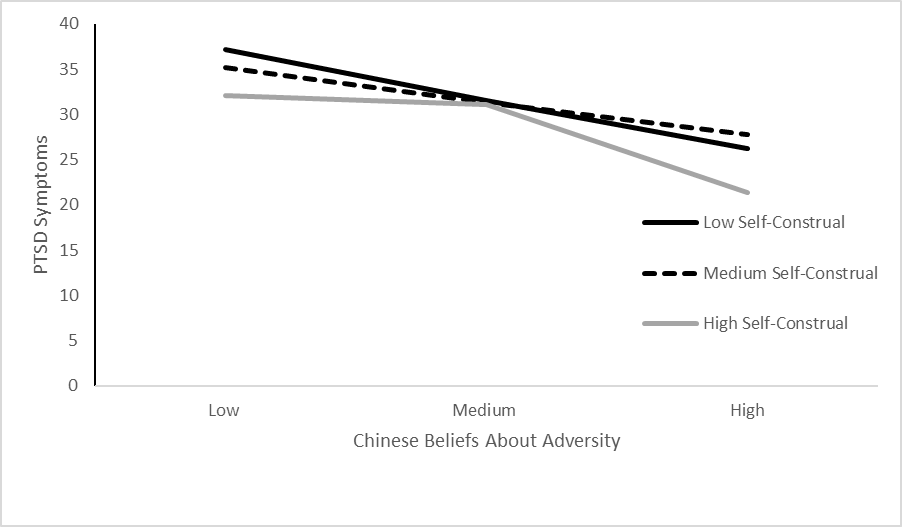
**

**Supplemental Figure 2**

*Moderating Effects of Self-Construal (Independent Reactive to Interdependent) on Secondary Control Appraisal and PTSD Symptoms*

**
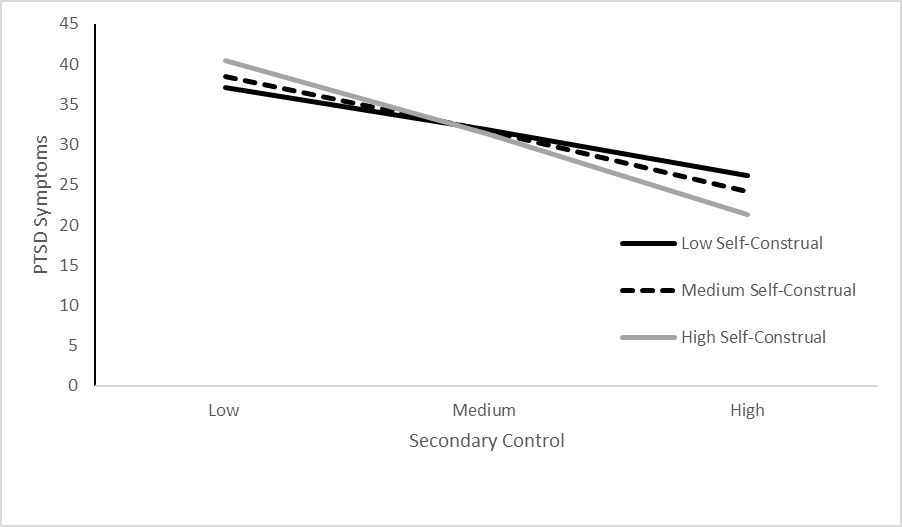
**
